# Circulating tumor cells: indicators of cancer progression, plasticity and utility for therapies

**DOI:** 10.3389/pore.2025.1612181

**Published:** 2025-10-20

**Authors:** Tamás Richárd Linkner, Zsófia Brigitta Nagy, Alexandra Kalmár, Eszter Farkas, Fruzsina Bányai, Nikolett Szakállas, István Takács, Béla Molnár

**Affiliations:** ^1^ Department of Internal Medicine and Oncology, Faculty of Medicine, Semmelweis University, Budapest, Hungary; ^2^ Department of Biological Physics, Faculty of Science, Eötvös Loránd University, Budapest, Hungary

**Keywords:** liquid biopsy, circulating tumor cells, cancer, EMT, senescence

## Abstract

Cancer is a deadly disease affecting millions of people worldwide. Circulating tumor cells (CTCs) represent a critical link between primary malignancies and metastasis, acting as key players in cancer dissemination, progression, and recurrence. Although rare, CTCs offer a valuable, non-invasive window into tumor biology and the evolution of disease in patients. CTCs can exist as single cells in the circulation, but some are shed and travel in larger groups, referred to as CTC clusters. These clusters possess a greater oncogenic potential compared to individual CTCs. In this review, we aim to provide insight into the dynamic biological processes underlying CTC generation, biology, and survival, with a focus on epithelial-to-mesenchymal transition (EMT) and beyond like cancer stem cells (CSCs), cellular plasticity, and senescence. A crucial aspect of CTC biology is EMT, a process that imparts cancer cells with increased motility, invasiveness, resistance to apoptosis, and the ability to intravasate and evade the immune system. Beyond EMT the cancer cells show further plasticity, allowing epithelial tumor cells to adopt mesenchymal or hybrid phenotypes, which enables adaptation to a changing microenvironment and enhances therapy resistance. Moreover, a subset of cancer cells can acquire stem cell-like properties, including self-renewal and tumor-initiating capacity. EMT, along with processes such as dedifferentiation, contributes to the generation of cancer stem cells. In recent years, studies have also highlighted the complex and paradoxical role of senescence in CTC biology. While senescence typically results in permanent cell cycle arrest, in cancer cells it may be reversible and can promote tumor cell dormancy, immune evasion, and metastatic reactivation. By exploring the connections between CTCs, EMT, CSCs, plasticity, and senescence, we aim to shed light on the unique biology of CTCs, their metastatic potential, and their contributions to tumor heterogeneity. We hope that a better understanding of these processes will help advance the development of novel biomarkers and therapeutic targets for solid tumors beyond EMT.

## Introduction

The aim of this review is to summarize recent advances regarding circulating tumor cells (CTCs), with a focus on their phenotype and plasticity. Moreover, we aim to shed light on the diagnostic properties of circulating tumor cells and cancer stem cells.

Cancer is one of the deadliest diseases affecting the human population, causing millions of deaths each year. In 2022 alone, there were 20 million newly reported cancer cases worldwide, with 9.7 million deaths. Breast carcinoma is the most common type of cancer in women (2.3 million new cases each year), while lung (1.5 million new cases each year) and prostate (1.4 million new cases each year) cancers are the most prevalent malignancies in men. Among both sexes, lung cancer is the most frequently diagnosed carcinoma with 2.5 million new cases each year [1–3].

Cancer is a disease caused by multiple mutations in a cell, leading to an altered cellular state. It is characterized by abnormal growth, spread, resource consumption, tissue disruption, and impairment of normal bodily functions. Environmental factors, viruses, bacteria, chemical agents, or radiation exposure can all contribute to cancer development [4, 5].

To fight an effective battle against cancer, understanding the disease, its progression, and developing new progression targeting therapeutic techniques is of utmost importance.

Our workgroup has previously conducted examinations of circulating tumor cells (CTCs) and CTC clusters. Using magnetic cell separation, we successfully detected cytokeratin (CK)-positive CTCs and CTC clusters in the blood of colorectal cancer patients. Additionally, our workgroup found cytokeratin positive cells in interaction with cytokeratin negative cells when investigating CTC clusters. This was the first time this was observed in colorectal carcinoma (CRC) patients. Moreover, we also observed that chemotherapy reduces the number of CTCs and clusters in the blood but does not eliminate them [6]. In another of our studies, we found that the higher the number of single CTCs in the circulation, the higher the number of epithelial cells in CTC clusters [5]. In the same study, we concluded that the number of CTC singlets, doublets, and clusters correlates with cytokeratin 20 (CK20) qPCR results from the blood of CRC patients [7]. Moreover, we have performed several liquid biopsy-based analyses on the blood of colorectal cancer patients to investigate the potential diagnostic and therapeutic implications of cell-free nucleic acids. We found that the level of cfDNA was higher in patients with non-metastatic CRC and metastatic CRC compared to individuals with remission or stable disease [8, 9].

In this review, we aim to gather the most recent information on CTCs. Furthermore, we seek to explore their unique plasticity and highlight the significance of CK + epithelial CTC clusters in circulation. Additionally, we provide an overview of the most up-to-date techniques for CTC detection, analysis, and their relation to therapy decisions.

## CTC biology and diagnostic utilization

The most lethal feature of cancer is metastasis—a process involving the invasion of distant parts of the body by cancer cells that “break away” from the primary tumor and enter the circulation. These cells are referred to as circulating tumor cells (CTCs). CTCs can travel through the bloodstream either as single cells or in clusters. CTC clusters are defined as groups of two or more CTCs with stable cell–cell junctions. Although clusters represent only a minority of CTCs found in circulation, they possess a higher metastatic potential than single CTCs. Moreover, in several cancer types, the presence of CTC clusters indicates a worse clinical outcome compared to single CTCs [10, 11]. It has been shown that in non-small cell lung cancer (NSCLC), the prevalence of CTC clusters increases with advanced cancer stages. However, no correlation was observed between the number of CTC clusters and the tumor type or stage in lung cancer indicating that cluster number may not distinguish between the most advanced disease stages. However, correlation between CTC number and prognosis was found in a meta-analysis which considered the presence of CTC but not their number or phenotype characterization [12–14].

Other than the blood stream, CTCs can also enter into the lymphatic circulation, where they can reach local lymph nodes and differentiate leading to metastases. Lymph-specific CTCs are usually non-immunogenic so they can avoid detection by the immune system, especially by cytotoxic T cells which helps them in their metastasis initiation [15–17].

Additionally, CTCs are also capable of perineural invasion (PNI), which is defined as an invasion in, around, and through the nerves. PNI is usually associated with poor clinical outcome and decreased survival in different cancer types including ductal adenocarcinoma, prostate cancer, gastric cancer, breast cancer, pancreatic ductal adenocarcinoma and colorectal carcinoma [18–20].

Since these cells are shed into the circulation, peripheral blood serves as an excellent source for the selection and analysis of CTCs. Over the past decade, multiple liquid biopsy techniques have been developed for CTC isolation and analysis. These methods can be categorized as either label-dependent or label-independent techniques. Label-dependent techniques rely on interactions between cell surface markers expressed on CTCs and specific antibodies. These antibodies can be fixed to the surface of magnetic particles or microfluidic chips to enable positive selection of CTCs from blood or negative depletion of white blood cells. These approaches typically target EpCAM, a surface protein commonly expressed on CTCs (Table 1). Amongst these techniques, currently the CellSearch system by Janssen Diagnostics is the only FDA approved method which utilizes EpCAM-coated ferrofluidic nanoparticles for CTC detection. Other commercially available label dependent methods are AdnaTest by Adnagen and MagSweeper by Illumina both of which are based on immunomagnetic capture of CTCs [21–23].

**TABLE 1:** Main differences between single CTCs, CTC clusters and cfDNA.

Attributes	Single CTC	CTC cluster	Cell free DNA
Composition	Single cancer cells	Multiple cancer cells, often in conjugation with stromal and/or immune cells	Short DNA fragments from necrotic/apoptotic tumor cells
Survival in circulation	Low	High	Low
EMT Traits	Mainly mesenchymal	Mainly epithelial	None
Metastatic potential	Low	High	None
Prognostic value	Moderate	High. Associated with poor prognosis	High. Early cancer detection
Markers	EpCAM, Vimentin, N-cadherin	CD44, OCT4, SOX2	Mutations specific for the originating tumor (EGFR, KRAS1, BRCA1/2)

Label-independent detection methods, on the other hand, are based on the physical properties of CTCs, such as size. Using filters with defined pore sizes, the typically larger CTCs can be separated from smaller blood cells. Gradient centrifugation can also be employed, where lower-density cells such as erythrocytes and polymorphonuclear leukocytes settle at the bottom, while higher-density mononuclear leukocytes and CTCs remain in the upper layers. Overall, methods based on physical properties are cost-effective and preserve cell viability well. However, these techniques are often inefficient, yield low purity, and lack specificity. ISET by Rarecells diagnostics and Parylene filter by Circulogix are both based on filter based isolation and enrichment platforms available for label-free detection. Other techniques are also on the market such as RosetteSep by STEMCELL technologies and OncoQuick by Greiner BioOne which are based on density gradient separation [21–23].

CTCs carry information about the originating tumor, making them highly valuable for clinical applications. CTC analysis can be used for early tumor detection, enabling treatment at an earlier, more manageable stage. Usually, the number of CTCs in early disease are low roughly ∼1/10^8^ peripheral blood mononuclear cells (PBMC), while in metastatic cancers their number is much higher at 1/10^5^–10^7^ PBMCs. Moreover, the presence of CTCs in the circulation provides prognostic information, aids in predicting disease outcomes, and helps guide treatment decisions. Furthermore, molecular characterization and genome sequencing of CTCs can provide valuable insights for the development of personalized treatments [23–26].

As a few examples, Baek et al. used the fluid-assisted separation technique (FAST) to enrich CTCs from the blood of healthy donors and CRC patients. They found that CTC counts were significantly higher in CRC patients compared to healthy volunteers. Notably, all patients with stage 4 CRC were positive for CTCs [27]. Dalum et al. utilized the CellSearch system to analyze the blood of CRC patients before surgery, and reported that the presence of CTCs prior to the operation was associated with a significant decrease in recurrence-free survival [28].

Cristofanilli and colleagues, in their study of metastatic breast cancer patients, reported that the presence of more than 5 CTCs per 7.5 mL of blood was associated with shorter median progression-free survival and overall survival compared to patients with fewer than 5 CTCs [29]. According to a metaanalysis by Jin et al., the detection of CTCs in circulation is associated with poor prognosis in small cell lung cancer (SCLC) patients compared to those with non-small cell lung cancer. Moreover, they found that epithelial CTCs predict worse outcomes than mesenchymal CTCs in lung cancer patients [30].

### CTC clusters

**FIGURE 1:**
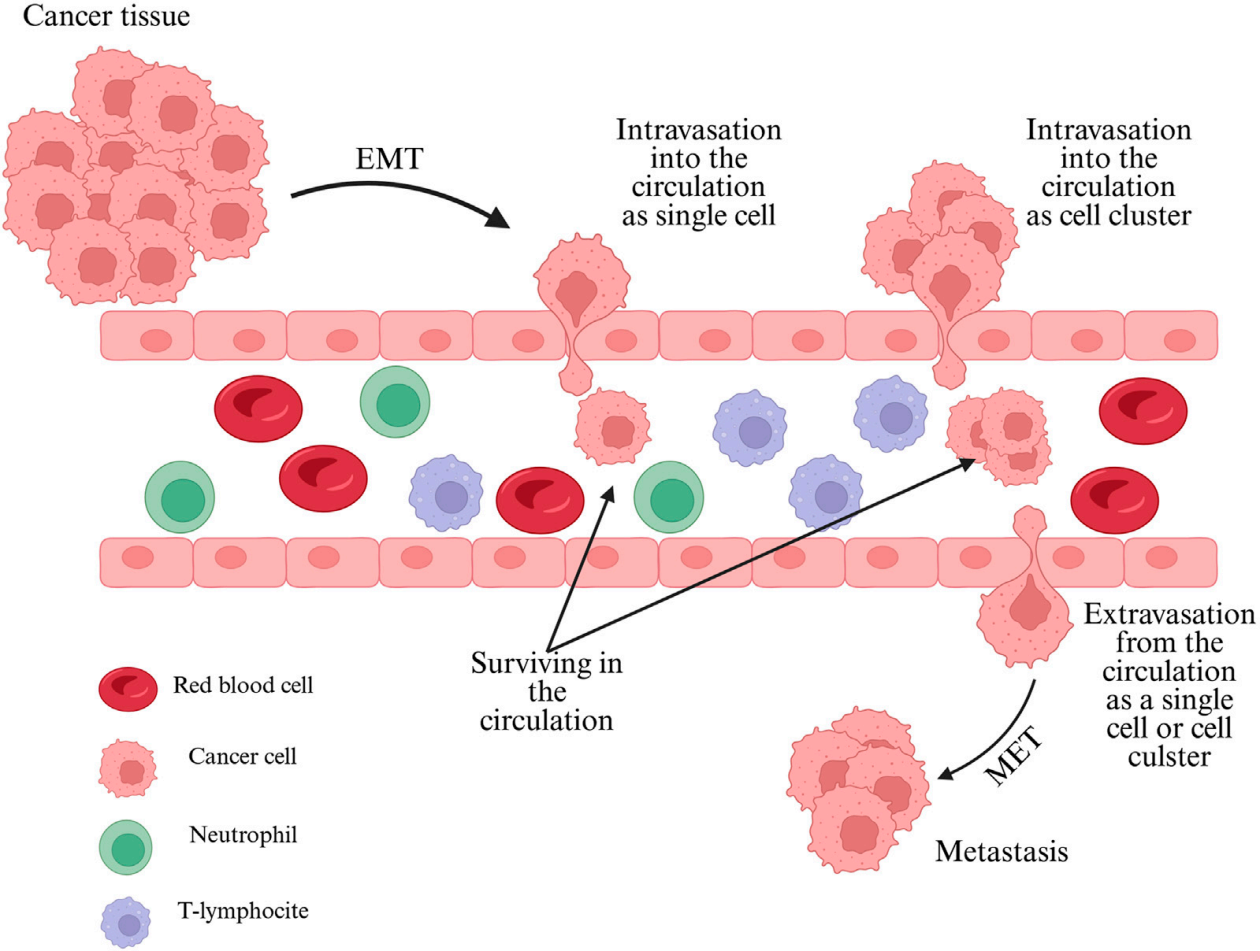
Main route of CTCs from the tumor to the secondary sites. Created in BioRender. Linkner, T. (2025) https://BioRender.com/jny6bvi.

CTCs can travel as single cells in the circulation; however, CTC clusters also exist, consisting of two or more CTCs attached together. These clusters can be homotypic, involving only CTCs, or heterotypic, when blood immune cells are also attached to CTCs [31]. Immune cells, such as neutrophils, can enhance the metastatic potential and survival of these clusters (Figure 1) [32].

Moreover, cancer associated fibroblast (CAF) which is a heterogenous network of cells originating from normal fibroblasts and cells like mesenchymal stem cells or endothelial cells can be found in heterotropic CTC clusters and they can increase the metastatic potential of the CTCs [33].

Compared to single CTCs, the larger size of clusters likely enhances their ability to adhere to the endothelium and promotes extravasation. Additionally, CTC clusters have been found to show increased expression of EMT/stemness markers such as CD44, OCT4, SOX2, Nanog, and SIM3A. They also exhibit elevated expression of cell junction proteins like plakoglobin and E-cadherin. Furthermore, the expression of markers that contribute to CTC aggregation, including KRT14, PAK2, and MUC1, is also upregulated (Table 1) [34].

It has been documented that the presence of circulating CTC clusters is associated with worse prognosis in various types of cancer. Additionally, CTC clusters may be protected from shear forces, anoikis, and immune surveillance while in circulation. Moreover, the metastatic potential of CTC clusters is significantly higher than that of single CTCs [35]. The main differences between CTCs, CTC clusters, and cell-free DNA are shown in Table 1.

### CTC heterogeneity

CTC heterogeneity can be divided into morphological and phenotypic heterogeneity of epithelial and mesenchymal cells in addition to tissue tumor heterogeneity which describes the genetic and somatic diversity within the primary tumor or between primary tumor and metastasises. Morphological heterogeneity refers to the different sizes and shapes that CTCs can take. This categorization also includes CTC clusters. In contrast, phenotypic heterogeneity refers to differences in gene expression patterns and cell surface markers [10, 36, 37].

The ability of CTCs to change their phenotype in response to environmental changes is referred to as CTC plasticity. One of the main expressions of CTC plasticity is a process called EMT [38]. This is the primary mechanism by which CTCs are formed. During EMT, epithelial tumor cells lose their adhesive ability and epithelial characteristics and acquire a mesenchymal phenotype, which results in mobile, highly metastatic CTCs. If they survive long enough in the circulation in the end they reach a distant organ, where CTCs undergo a reverse process known as mesenchymal-to-epithelial transition (MET) [23]. The ability of CTCs to transition back and forth between these cell states is referred to as EMT plasticity [39].

## Epithelial to mesenchymal transition

**FIGURE 2:**
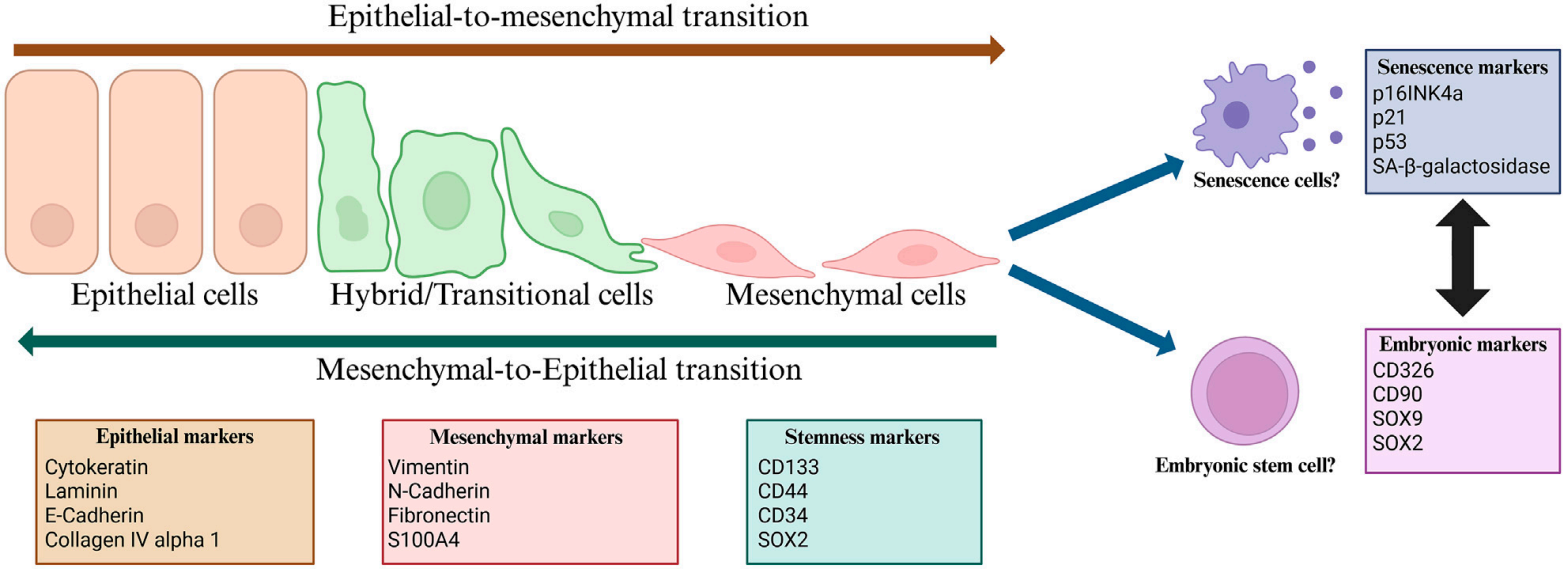
Basic overview of the process of EMT with basic markers and the possible stages after the mesenchymal state. Created in BioRender. Linkner, T. (2025) https://BioRender.com/tp6wqbm.

EMT is a complex process involving many molecular and cellular changes, such as the downregulation of epithelial markers (e.g., cytokeratins, E-cadherin, and claudins) and the upregulation of mesenchymal proteins (e.g., vimentin, N-cadherin, and fibronectin), which increase the mobility and invasiveness of the cell. The changes observed during EMT are regulated by transcription factors known as EMT-inducing transcription factors (EMT-TFs), such as Snail-1, Snail-2 (Slug), ZEB1, and Twist (Figure 2) [40]. It is widely accepted that the process of EMT generates multiple hybrid phenotypes along the epithelial-mesenchymal axis, contributing to tumor heterogeneity. Both epithelial and mesenchymal states are believed to harbor limited metastatic potential; however, certain hybrid phenotypes can possess a higher degree of EMT plasticity, enabling them to survive and adapt to different microenvironments encountered during metastatic spread [40].

The process leading to metastasis is complex, involving several biological steps. First, metastatic cells must undergo EMT, detach from the primary tumor, invade the bloodstream, survive in circulation, disseminate into distant organs, extravasate, undergo MET, colonize, and form micrometastasis. Only a fraction of CTCs are capable of undergoing metastatic transformation; these cells are referred to in the literature as circulating cancer stem cells [41].

Balcik-Ercin et al. found in their colorectal carcinoma-derived CTC cell line, that the expression of SIX1, an EMT marker important for the mesenchymal profile, was downregulated. This suggests that tumor cells can utilize alternative pathways to activate genes that promote their plasticity and invasiveness. Furthermore, they found that the MET transcription factor GRHL2 was overexpressed in their CTC lines. GRHL2 may stabilize the epithelial-mesenchymal hybrid phenotype and support cell migration [38].

Seo et al. investigated the phenotypic heterogeneity of CTCs in SCLC using assays to characterize rare cells. In an EpCAM-targeted assay, utilizing a variety of biomarkers, they observed a wide range of CK and EpCAM expression in the CTC population. Their single-cell sequencing results reinforce the presence of tumor cell plasticity by indicating that a phenotypically heterogeneous population of cells can be genomically stable. Recent evidence suggests that cancer cells exhibit a hybrid mesenchymal and epithelial character, and this plasticity is associated with their metastatic ability and poor patient prognosis [42].

It has been described that the activation of the EMT program does not always result in a fully mesenchymal phenotype. It is likely that a partial EMT status is achieved in both non-transformed and cancer cells, the resulting hybrid cells carry both epithelial and mesenchymal markers. Moreover, these hybrid cells are more likely to acquire stemness [33]. Indeed, it has been shown that with EMT induction, breast cancer cells can acquire cancer stem cell markers, such as CD44 [43, 44].

The fact that stemness markers can be expressed by cells undergoing EMT opens the possibility that differentiated mesenchymal cells can also acquire stemness characteristics, leading to the formation of new mesenchymal stem cells.

## Cancer stem cells

There are multiple therapies that can be implemented to treat cancer, such as radiotherapy, surgery, and chemotherapy. However, cancer cells can develop resistance to chemotherapy, which is a major factor in therapy failure and poor patient survival [45, 46].

Due to the stress generated by the changing environment and therapy, genetic mutations occur in cancer cells, leading to cancer heterogeneity and, in turn, therapy resistance. Heterogeneity among patients due to environmental, somatic, and germline factors is called intertumoral, while uneven distributions of genetically diverse subpopulations of cancer cells in the same tumor are referred as intratumoral heterogeneity. Moreover, the differences between a primary tumor and the metastasis in a patient are also called intertumoral heterogeneity [47–49].

One of the factors contributing to intratumoral heterogeneity is the presence of cancer stem cells (CSCs), a subset of cancer cells possessing stem cell characteristics such as self-renewal and the ability to differentiate [50, 51]. CSCs were first identified in acute myeloid leukemia (AML) after transplanting isolated CD34+/CD38− cancer cells into non-obese diabetic/severe combined immunodeficient (NOD/SCID) mice. Since then, CSCs have been described in a variety of hematological and solid tumors, such as pancreatic, breast, and colon malignancies [52]. The origin of CSCs is highly debated, with multiple hypotheses suggesting that they arise from either adult stem cells, mutated adult progenitor cells, or cancer cells that gain stem-like properties through dedifferentiation. CSCs can be separated from normal stem cells via the expression of specific cell surface markers such as CD133, CD24, CD44, epithelial cell adhesion molecule (EpCAM), and CD200. Moreover, intracellular proteins have also been used as markers of CSCs, such as aldehyde dehydrogenase 1 (ALDH1) (Figure 2) [53–55].

There’s a connection between the previously mentioned EMT and cancer stemness. The expression of EMT inducing transcription factors such as ZEB1, SNAIL1 and 2 by cancer cells initiates the expression of stem cell markers SOX2, BMI1 and OCT4. It is described that mesenchymal and stemness traits, characterise cancer stem cells within the tumor mass. This indicates that CSCs have specific abilities similar to embryonic stem cells [56].

## Cancer senescence

Chemo- and radiotherapy induce DNA damage in differentiated cancer cells, which in turn leads to therapy-induced senescence (TIS). Senescence is a cell state characterized by prolonged cell-cycle arrest, enhanced secretory capacity, macromolecular damage, and altered metabolism. The main defining characteristic of senescence is stable growth arrest, which ensures that damaged or transformed cells do not preserve and perpetuate their genomes. During this process, specific molecular markers are activated, such as p16INK4a/Rb and p53/p21CIP1. Senescence also has physiological roles; the process is triggered in response to damage and allows the suppression of potentially dysfunctional, transformed, or aged cells. However, the aberrant accumulation of senescent cells during aging has potential detrimental effects, such as contributing to renal dysfunction and type II diabetes (Figure 2) [57].

The senescent state of cancer cells can be beneficial as these cells induce inflammation and attract immune cells, which clear the senescent cancer cells. One of the main characteristics of senescent cells is the senescence-associated secretory phenotype (SASP). They secrete interleukins and other ligands that can negatively affect cancer initiation and progression.

However, the previously mentioned TIS also induces cancer remodeling and promotes CSC generation. Moreover, senescent tumor cells can cause changes in the tumor microenvironment, further promoting cancer development. SASP can also provide a positive environment for tumor progression. It was shown that SASP components can promote cancer cell growth, invasion, metastasis, and tumor vascularization [54, 58, 59].

Cancer cells can escape the senescent state through the acquisition of genetic and epigenetic features, which make them plastic. Additionally, via the paracrine action of SASP, cells in close proximity to tumor cells can be imparted with tumorigenic capacities. Senescence escape and cellular reprogramming via SASP are essential components of epithelial tumor progression. Tumor cells achieve the previously mentioned plasticity through the initiation of EMT [60].

### Polyploid senescence cells

Polyploid cells are large, multicellular entities formed by cell fusion and/or endoreduplication [61]. In the case of cancer, polyploid giant cancer cells arise due to genotoxic stress caused by chemo and/or radiotherapy. They mostly exhibit features of senescence, and they also give rise to aneuploid or diploid daughter cells, which can undergo mitosis. This might be responsible for the heterogeneous nature of cancer cells. Additionally, they can secrete an array of cytokines, chemokines, and growth factors which influences the tumor microenvironment and contributes to poor prognosis like therapy resistance [62, 63]. Polyploid tumor cells are able to differentiate into different types of cells, including adipose tissue or bone, which indicates that these cells possess cancer stem cell properties [64]. Like senescence, polyploidy can develop in response to therapy. The connection between senescence, polyploidy, and therapy has been observed in multiple cancer types. Cancer cells, when exposed to DNA-damaging agents, develop polyploidy upon entering senescence. Senescent polyploid cells are involved in the generation of proliferating progeny cells. This likely occurs through depolyploidization, during which mononucleated daughter cells are created from the multinucleated tumor, either by budding or asymmetric cell division. Depolyploidization can be a way for cancer cell to escape senescence [65].

## Circulating tumor cells and circulating DNA an overlap is to find

Liquid biopsy-based monitoring of cancer is a promising, non-invasive method which usually involves blood or urine collection, followed by the analysis of extracellular vesicles, circulating tumor cells (CTCs), or circulating tumor DNA (ctDNA) [66].

ctDNA is a form of nucleic acid released mainly from apoptotic or necrotic tumor cells into the circulation. In the peripheral blood, ctDNA circulates in the form of nucleosomes, which can be isolated, and their genetic and epigenetic properties can be analyzed to provide information about the originating tumor (Table 1) [67].

A few examples are listed below for the utilization and shortcomings of CTCs and ctDNA in the diagnosis of different epithelial cancers. Both CTCs and ctDNA can be used in the early detection of colorectal cancer and can be used in prognosis and treatment response monitoring, as well [68]. However, the level of CTCs is usually low in CRC patients, especially in the early phase of the disease. On the other hand, ctDNA can be detected more easily and provide real-time molecular information to monitor treatment response and relapse [69].

In early stage breast cancer, CTCs are present in low numbers and difficult to analyze, while ctDNA is more readily detectable and useful for monitoring tumor response, drug resistance, and mutations. In metastatic breast cancer, ctDNA efficiently tracks treatment response and tumor heterogeneity, whereas elevated CTC levels serve as prognostic markers [70].

CTCs are more common in small cell lung cancer (SCLC) than in non-small cell lung cancer (NSCLC) [71]. Despite this fact, in NSCLC, CTCs provide more informative mutation detection than ctDNA because of more sensitive genotyping [72]. However, as mentioned before CTC counts are highest in SCLC due to rapid tumor growth and early spread, making them better prognostic markers than ctDNA in this subtype [73]. In NSCLC, both CTCs and ctDNA can serve as diagnostic, prognostic, and therapeutic monitoring tools [74].

Our workgroup previously carried out experiments where with high sensitivity we detected the septin 9 gene (*SEPT9*) from circulation which is an excellent marker of CRC [75]. Moreover in a separate study we also detected that compared to healthy tissue, SEPT9 is hypermethylated in adenoma and CRC cells. Our results indicated that changes in the SEPT9 methylation reflects the cellular progression towards malignancy in the colon mucosa [76]. A list of biomarkers which can be detected with ctDNA analysis are shown on Table 2 with relevant mutations and associated cancers.

**TABLE 2:** Examples of biomarkers which can be detected with ctDNA analysis and their clinical utility and prognostic relevance with the most common mutations in associated cancer.

Gene	Associated cancer	Mutations in associated cancer	Prognostic/therapeutic relevance in associated cancer	Clinical utility in associated cancer	Source
TP53	Ovarian, head and neck, breast	R175H, R248Q	Poor prognosis	Prognosis and therapy prediction	[77–82]
EGFR	Lung, colon	L858R, T790M, C797S	Therapy prediction monitoring	Therapy selection, disease monitoring	[83–89]
KRAS	Pancreatic, lung, colon	G12D, G12V, G12C	Response to inhibitors	Disease and treatment monitoring	[90–95]
BRAF V600E	Melanoma, colon	V600E, V600K	Response to inhibitors	Treatment monitoring, survival prediction	[96–99]
PIK3CA	Breast, colon, endometrial	H1047R, E545K	Poor prognosis	Survival prediction	[100–106]
SEPT9	Colon	Methylation in the promoter region	Poor prognosis	Early diagnosis, survival prediction	[75, 107–109]
BRCA1/2	Ovarian, breast	Frameshift, splicing mutations	Response to inhibitors	Survival prediction	[110–112]
HER2	Breast	S310F, L755S	Poor prognosis	Treatment and relapse monitoring	[113–116]
CTNNB1	Liver	S45F, D32Y	Prognostic indicator	Treatment and tumor dynamics monitoring	[117–120]

In a study, Kong et al. found mutations in CTCs and ctDNA that matched those in the primary tumor. They also discovered that the top mutated genes in CTCs and ctDNA had prognostic value when applied to existing cohorts of cancer [121].

Koyanagi et al. also found in their research that, in stage IV melanoma patients, the number of CTCs correlated with the methylation of ctDNA molecules [122]. Additionally, in the peripheral blood of breast cancer patients, the level of ctDNA correlated with the presence of CTCs. This potentially suggests that CTCs are a major source of ctDNA, or that high numbers of CTCs and ctDNA are both features of a more aggressive tumor [123]. This correlation between ctDNA and CTCs was also observed in another study. Furthermore, methylated ctDNA and CTCs correlated with aggressive tumor biology and advanced disease [124].

### Therapy of minimal residual disease (MRD), cancer relapse based on circulating tumor cells

MRD is defined as a small number of cancer cells that remain in the body after treatment and can cause disease relapse [66].

For the tumor to detoriate, many pathophysiological cascades are required, such as the loss of cellular adhesion, increased cancer motility, invasiveness, entry into and survival in the circulation, and extravasation into the surrounding tissue. Circulating tumor cells (CTCs) represent an important phase in these processes [125, 126].

Liquid biopsy-based methods are non-invasive and provide an accurate method for monitoring the stage of the tumor. Before surgery, CTCs are much more informative about the tumor and correlates with disease stage compared to ctDNA [69, 126, 127]. However, ctDNA is much better at monitoring therapy and relapse as it is an accurate real-time biomarker of solid tumors and also a method to analyze MRD [69, 128]. Additionally ctDNA detection in the circulation of postoperative patients has a 100% possibility of predicting tumor relapse [129]. Furthermore, in a study, Radovich and colleagues found that the presence of ctDNA and CTCs after neoadjuvant chemotherapy correlates with cancer relapse in triple-negative breast cancer. A part of the observed patient group were positive for one marker, such that the sensitivity for recurrence detection went from 79% with ctDNA alone and 62% with CTC alone to 90% when combined [130].

Moreover, CTC detection and analysis also provide information about MRD and late-stage recurrence. In colorectal cancer (CRC) patients, CTC positivity before surgery significantly reduces overall survival (OS) and progression-free survival (PFS) compared to CTC-negative patients. Additionally, CTCs can be used as independent prognostic indicators of PFS and OS in advanced CRC. Furthermore, there are differences between the subtypes of CTCs. Mesenchymal-type CTCs are predominantly found in patients with metastatic CRC [130].

In the last few years, immune checkpoint therapies gained huge attention in the treatment of cancer. These methods are based on the inhibition of immune cell inactivating signals generated by cancer cells through cell surface molecules like PD-1 or CTLA-4 [131]. Most of the CTCs are eliminated by the immune system, however a subset of cells can evade the immune surveillance through various ways. One of the escape mechanisms are based on plasticity. For example, through epithelial-to-mesenchymal transition, cancer cells can increase PD-L1 expression on their surface, induce regulatory T cells, or inhibit dendritic cell functions, all of which helps them evade the immune system. In the light of this information, targeting tumor cell plasticity can sensitize cancer cells to immune-mediated cell death [132, 133].

### Adaptive cancer therapy based on cancer cell plasticity

Tumor cell plasticity is a non-mutational process that contributes to drug resistance. Plasticity includes the reactivation of developmental programs such as epithelial-to-mesenchymal transition (EMT), acquisition of cancer stem cell properties, and trans-differentiation [134]. Plasticity provides the tumor with the ability to shift between different states, from low tumorigenic potential to an undifferentiated cancer stem cell-like state [135]. Alterations in the cancer state are caused by changes in the tumor microenvironment, genetic or epigenetic changes, or selective pressure from treatment. There is also evidence suggesting that cancer cells have intrinsic plasticity, which helps the tumor adapt to the changing microenvironment. This flexibility in the cell state may contribute to therapy resistance [136]. It was described that CTCs with stem or mesenchymal characteristics are more aggressive and less susceptible to chemotherapy in case of breast cancer [137]. EMT which is associated with the increased invasiveness of tumor cells are also involved in the generation of resistance mechanisms. Inhibition of EMT was shown to reduce chemotherapy resistance [138]. Moreover, it was described that inhibition of EMT transcription factors can reduce cancer stem cells [139]. In case of senescence, it was observed that transcription factors which promote EMT can reduce senescence in cancer cells. However the mechanisms behind this process are not yet fully understood [140, 141].

Additionally, numerous factors are involved in the cancer plasticity-mediated therapy resistance, such as transcription factors like SOX2 or ZEB1 [142, 143]. Epigenetic modifications, such as DNA methylation, are also significant factors in therapy resistance [144]. Indeed, Caamano et al found that methylation in CTCs were associated with changes in gene expression which contributes to CTC therapy resistance [145].

Strategies to combat plasticity-induced therapy resistance can be categorized as: prevention of the emergence of plasticity, selective elimination of emerging therapy-resistant plastic cells, and reversion of the phenotypic switch [136].

Cancer therapy has the potential to initiate the creation of a therapy-resistant cancer cell population. Cancer is highly heterogeneous, while therapy is often administered in a linear, strict manner [146]. Meanwhile, adaptive therapy employs a treatment strategy based on tumor evolution. After treatment, the tumor is different compared to its pre-treatment state, which means that the following treatment should be applied differently. Adaptive therapy needs to adjust treatment strategies in light of the changing tumor [147].

## Conclusion

CTCs are a pivotal and critical point in the progression and understanding of cancer especially metastasis. Their presence in the circulation of the patient either as single cells or clusters highlights their important role in cancer dissemination. Due to their unique ability to mirror the genetic characteristics of the originating tumor CTCs provide valuable, minimally invasive means of accessing real-time information about the biology of the tumors. However, due to changes like EMT, CTCs can diverge phenotypically from the original tumor. As EMT usually activated in tumor cell sub-populations during dissemination CTCs carry the phenotypic information of the originating cell population. Despite, it has been demonstrated that CTCs carry prognostic and diagnostic utility in detecting MRD, guiding therapeutic decisions and monitoring relapse especially when utilized alongside cfDNA.

Looking forward, advances in CTC isolation and characterization techniques may provide a way for more precise and personalized therapy. With the integration of multi-omics approaches like single-cell sequencing and artificial intelligence researchers could further enhance the ability to profiling these rare cells and also offer deeper insights into the evolution of the tumor and its resistance to therapy.

Finally, leveraging CTCs in clinical practice holds promise for early detection and better monitoring and also for targeted treatment development which could transform and improve cancer care and ultimately patient outcomes. When used in conjunction with ctDNA CTCs can provide a more comprehensive view of tumor dynamics as ctDNA offers insight into genetic alterations while CTCs allow phenotypic and functional analyses. However, there are still challenges remain before CTC-based approaches can be fully utilized in routine clinical use. These include the extremely low abundance of CTCs in early-stage disease compared to ctDNA, limitations in current isolation and detection technologies, and the lack of standardized protocols across platforms. Furthermore, heterogeneity among CTCs, including variable expression of surface markers due to processes like EMT, can lead to false negatives and complicate interpretation. Addressing these technical and biological hurdles through continued innovation and validation in large clinical studies will be important for fully realizing the potential of CTCs in precision oncology.

## References

[1] World Health Organization. Global cancer burden growing, amidst mounting need for services (2024). Available online at: https://www.who.int/news/item/01-02-2024-global-cancer-burden-growing--amidst-mounting-need-for-services (Accessed January 31, 2025). PMC11115397 38438207

[2] Bray F Laversanne M Sung H Ferlay J Siegel RL Soerjomataram I Global cancer statistics 2022: GLOBOCAN estimates of incidence and mortality worldwide for 36 cancers in 185 countries. CA: a Cancer J clinicians (2024) 74(3):229–63. 10.3322/caac.21834 38572751

[3] World cancer research Fund. Worldwide cancer data (2024). Available online at: https://www.wcrf.org/preventing-cancer/cancer-statistics/worldwide-cancer-data/ (Accessed July 20, 2025).

[4] Brown J Amend SR Austin RH Gatenby RA Hammarlund EU Pienta KJ. Updating the definition of cancer. Mol Cancer Res (2023) 21(11):1142–7. 10.1158/1541-7786.MCR-23-0411 37409952 PMC10618731

[5] Seyed HH Mohammadamin D. Review of cancer from perspective of molecular. J Cancer Res Pract (2017) 4(4):127–9. 10.1016/j.jcrpr.2017.07.001

[6] Molnar B Ladanyi A Tanko L Sréter L Tulassay Z. Circulating tumor cell clusters in the peripheral blood of colorectal cancer patients. Clin Cancer Res official J Am Assoc Cancer Res (2001) 7(12):4080–5. 11751505

[7] Molnar B Floro L Sipos F Toth B Sreter L Tulassay Z. Elevation in peripheral blood circulating tumor cell number correlates with macroscopic progression in UICC stage IV colorectal cancer patients. Dis markers (2008) 24(3):141–50. 10.1155/2008/941509 18334735 PMC3850608

[8] Galamb O Barták BK Kalmár A Nagy ZB Szigeti KA Tulassay Z Diagnostic and prognostic potential of tissue and circulating long non-coding RNAs in colorectal tumors. World J Gastroenterol (2019) 25(34):5026–48. 10.3748/wjg.v25.i34.5026 31558855 PMC6747286

[9] Barták B Fodor T Kalmár A Nagy ZB Zsigrai S Szigeti KA A liquid biopsy-based approach for monitoring treatment response in post-operative colorectal cancer patients. Int J Mol Sci (2022) 23(7):3774. 10.3390/ijms23073774 35409133 PMC8998310

[10] Aceto N Bardia A Miyamoto DT Donaldson MC Wittner BS Spencer JA Circulating tumor cell clusters are oligoclonal precursors of breast cancer metastasis. Cell (2014) 158(5):1110–22. 10.1016/j.cell.2014.07.013 25171411 PMC4149753

[11] Castro-Giner F Aceto N. Tracking cancer progression: from circulating tumor cells to metastasis. Genome Med (2020) 12(1):31. 10.1186/s13073-020-00728-3 32192534 PMC7082968

[12] Hong Y Fang F Zhang Q. Circulating tumor cell clusters: what we know and what we expect. Int J Oncol (2016) 49(6):2206–16. (Review). 10.3892/ijo.2016.3747 27779656 PMC5117994

[13] el al M. Prevalence and number of circulating tumour cells and microemboli at diagnosis of advanced NSCLC. J Cancer Res Clin Oncol (2026) 142(1):195–200. 10.1007/s00432-015-2021-3 PMC11819433 26210156

[14] Wang J Huang J Wang K Xu J Huang J Zhang T. Prognostic significance of circulating tumor cells in non-small-cell lung cancer patients: a meta-analysis. PloS one (2013) 8(11):e78070. 10.1371/journal.pone.0078070 24223761 PMC3817175

[15] Mohammed SI Torres-Luquis O Walls E Lloyd F. Lymph-circulating tumor cells show distinct properties to blood-circulating tumor cells and are efficient metastatic precursors. Mol Oncol (2019) 13(6):1400–18. 10.1002/1878-0261.12494 31026363 PMC6547792

[16] Ubellacker JM Tasdogan A Ramesh V Shen B Mitchell EC Martin-Sandoval MS Lymph protects metastasizing melanoma cells from ferroptosis. Nature (2020) 585(7823):113–8. 10.1038/s41586-020-2623-z 32814895 PMC7484468

[17] Leong SP Naxerova K Keller L Pantel K Witte M. Molecular mechanisms of cancer metastasis via the lymphatic versus the blood vessels. Clin & Exp metastasis (2022) 39(1):159–79. 10.1007/s10585-021-10120-z 34767139 PMC8967809

[18] Wang H Huo R He K Cheng L Zhang S Yu M Perineural invasion in colorectal cancer: mechanisms of action and clinical relevance. Cell Oncol (Dordrecht, Netherlands) (2024) 47(1):1–17. 10.1007/s13402-023-00857-y 37610689 PMC10899381

[19] Bahmad HF Wegner C Nuraj J Avellan R Gonzalez J Mendez T Perineural invasion in breast cancer: a comprehensive review. Cancers (2025) 17(12):1900. 10.3390/cancers17121900 40563551 PMC12190579

[20] Sun Y Jiang W Liao X Wang D. Hallmarks of perineural invasion in pancreatic ductal adenocarcinoma: new biological dimensions. Front Oncol (2024) 14:1421067. 10.3389/fonc.2024.1421067 39119085 PMC11307098

[21] Habli Z AlChamaa W Saab R Kadara H Khraiche ML. Circulating tumor cell detection technologies and clinical utility: challenges and opportunities. Cancers (2020) 12(7):1930. 10.3390/cancers12071930 32708837 PMC7409125

[22] Ju S Chen C Zhang J Xu L Zhang X Li Z Detection of circulating tumor cells: opportunities and challenges. Biomarker Res (2022) 10(1):58. 10.1186/s40364-022-00403-2 35962400 PMC9375360

[23] Lin D Shen L Luo M Zhang K Li J Yang Q Circulating tumor cells: biology and clinical significance. Signal Transduction Targeted Therapy (2021) 6(1):404. 10.1038/s41392-021-00817-8 34803167 PMC8606574

[24] Salu P Reindl KM. Advancements in circulating tumor cell research: bridging biology and clinical applications. Cancers (2024) 16(6):1213. 10.3390/cancers16061213 38539545 PMC10969710

[25] Heidrich I Abdalla TSA Reeh M Pantel K. Clinical applications of circulating tumor cells and circulating tumor DNA as a liquid biopsy marker in colorectal cancer. Cancers (2021) 13(18):4500. 10.3390/cancers13184500 34572727 PMC8469158

[26] Allan AL Keeney M. Circulating tumor cell analysis: technical and statistical considerations for application to the clinic. J Oncol (2010) 2010:426218. 10.1155/2010/426218 20049168 PMC2798617

[27] Baek DH Kim GH Song GA Han IS Park EY Kim HS Clinical potential of circulating tumor cells in colorectal cancer: a prospective study. Clin translational Gastroenterol (2019) 10(7):e00055. 10.14309/ctg.0000000000000055 31246593 PMC6708664

[28] van Dalum G Stam GJ Scholten LFA Mastboom WJB Vermes I Tibbe AGJ Importance of circulating tumor cells in newly diagnosed colorectal cancer. Int J Oncol (2015) 46(3):1361–8. 10.3892/ijo.2015.2824 25572133

[29] Cristofanilli M Budd GT Ellis MJ Stopeck A Matera J Miller MC Circulating tumor cells, disease progression, and survival in metastatic breast cancer. The New Engl J Med (2004) 351(8):781–91. 10.1056/NEJMoa040766 15317891

[30] Jin F Zhu L Shao J Yakoub M Schmitt L Reißfelder C Circulating tumour cells in patients with lung cancer universally indicate poor prognosis. Eur Respir Rev official J Eur Respir Soc (2022) 31(166):220151. 10.1183/16000617.0151-2022 36517047 PMC9879327

[31] Schuster E Taftaf R Reduzzi C Albert MK Romero-Calvo I Liu H. Better together: circulating tumor cell clustering in metastatic cancer. Trends Cancer (2021) 7(11):1020–32. 10.1016/j.trecan.2021.07.001 34481763 PMC8541931

[32] Chen Q Zou J He Y Pan Y Yang G Zhao H A narrative review of circulating tumor cells clusters: a key morphology of cancer cells in circulation promote hematogenous metastasis. Front Oncol (2022) 12:944487. 10.3389/fonc.2022.944487 36059616 PMC9434215

[33] Hurtado P Martínez-Pena I Piñeiro R. Dangerous liaisons: circulating tumor cells (CTCs) and cancer-associated fibroblasts (CAFs). Cancers (2020) 12(10):2861. 10.3390/cancers12102861 33027902 PMC7599894

[34] Bates M Mohamed BM Ward MP Kelly TE O'Connor R Malone V Circulating tumour cells: the good, the bad and the ugly. Biochim Biophys Acta Rev Cancer (2023) 1878(2):188863. 10.1016/j.bbcan.2023.188863 36796527

[35] Yang Y Huang G Lian J Long C Zhao B Liu X Circulating tumour cell clusters: isolation, biological significance and therapeutic implications. BMJ Oncol (2024) 3(1):e000437. 10.1136/bmjonc-2024-000437 39886139 PMC11557725

[36] Wang Q Tan LM. Advances in the role of circulating tumor cell heterogeneity in metastatic small cell lung cancer. Cancer innovation (2023) 3(2):e98. 10.1002/cai2.98 38946931 PMC11212323

[37] Menyailo ME Tretyakova MS Denisov EV. Heterogeneity of circulating tumor cells in breast cancer: identifying metastatic seeds. Int J Mol Sci (2020) 21(5):1696. 10.3390/ijms21051696 32121639 PMC7084665

[38] Balcik-Ercin P Cayrefourcq L Soundararajan R Mani SA Alix-Panabières C. Epithelial-to-Mesenchymal plasticity in circulating tumor cell lines sequentially derived from a patient with colorectal cancer. Cancers (2021) 13(21):5408. 10.3390/cancers13215408 34771571 PMC8582537

[39] Jie XX Zhang XY Xu CJ. Epithelial-to-mesenchymal transition, circulating tumor cells and cancer metastasis: mechanisms and clinical applications. Oncotarget (2017) 8(46):81558–71. 10.18632/oncotarget.18277 29113414 PMC5655309

[40] Genna A Vanwynsberghe AM Villard AV Pottier C Ancel J Polette M EMT-associated heterogeneity in circulating tumor cells: sticky friends on the road to metastasis. Cancers (2020) 12(6):1632. 10.3390/cancers12061632 32575608 PMC7352430

[41] Yang MH Imrali A Heeschen C. Circulating cancer stem cells: the importance to select. Chin J Cancer Res = Chung-kuo yen Cheng yen chiu (2015) 27(5):437–49. 10.3978/j.issn.1000-9604.2015.04.08 26543330 PMC4626824

[42] Seo J Kumar M Mason J Blackhall F Matsumoto N Dive C Plasticity of circulating tumor cells in small cell lung cancer. Scientific Rep (2023) 13(1):11775. 10.1038/s41598-023-38881-5 37479829 PMC10362013

[43] Verstappe J Berx G. A role for partial epithelial-to-mesenchymal transition in enabling stemness in homeostasis and cancer. Semin Cancer Biol (2023) 90:15–28. 10.1016/j.semcancer.2023.02.001 36773819

[44] Mani SA Guo W Liao MJ Eaton EN Ayyanan A Zhou AY The epithelial-mesenchymal transition generates cells with properties of stem cells. Cell (2008) 133(4):704–15. 10.1016/j.cell.2008.03.027 18485877 PMC2728032

[45] Morel AP Lièvre M Thomas C Hinkal G Ansieau S Puisieux A. Generation of breast cancer stem cells through epithelial-mesenchymal transition. PloS one (2008) 3(8):e2888. 10.1371/journal.pone.0002888 18682804 PMC2492808

[46] Debela DT Muzazu SG Heraro KD Ndalama MT Mesele BW Haile DC New approaches and procedures for cancer treatment: current perspectives, SAGE Open Med (2021), 9: 20503121211034366. 10.1177/20503121211034366 34408877 PMC8366192

[47] Lei Z Tian Q Teng QX Wurpel JND Zeng L Pan Y Understanding and targeting resistance mechanisms in cancer. MedComm (2023) 4(3):e265. 10.1002/mco2.265 37229486 PMC10203373

[48] Khan SU Fatima K Aisha S Malik F. Unveiling the mechanisms and challenges of cancer drug resistance. Cell Commun signaling: CCS (2024) 22(1):109. 10.1186/s12964-023-01302-1 38347575 PMC10860306

[49] Dagogo-Jack I Shaw AT. Tumour heterogeneity and resistance to cancer therapies. Nat Rev Clin Oncol (2018) 15(2):81–94. 10.1038/nrclinonc.2017.166 29115304

[50] MacDonald WJ Purcell C Pinho-Schwermann M Stubbs NM Srinivasan PR El-Deiry WS. Heterogeneity in cancer. Cancers (2025) 17(3):441. 10.3390/cancers17030441 39941808 PMC11816170

[51] Chang JC. Cancer stem cells: role in tumor growth, recurrence, metastasis, and treatment resistance. Medicine (2016) 95(1Suppl. 1):S20–5. 10.1097/MD.0000000000004766 27611935 PMC5599212

[52] Prasetyanti PR Medema JP. Intra-tumor heterogeneity from a cancer stem cell perspective. Mol Cancer (2017) 16(1):41. 10.1186/s12943-017-0600-4 28209166 PMC5314464

[53] Bu Y Cao D. The origin of cancer stem cells. Front Biosci (Scholar edition) (2012) 4(3):819–30. 10.2741/s302 22202093

[54] Walcher L Kistenmacher AK Suo H Kitte R Dluczek S Strauß A Cancer stem cells-origins and biomarkers: perspectives for targeted personalized therapies. Front Immunol (2020) 11:1280. 10.3389/fimmu.2020.01280 32849491 PMC7426526

[55] Phi LTH Sari IN Yang YG Lee SH Jun N Kim KS Cancer stem cells (CSCs) in drug resistance and their therapeutic implications in cancer treatment. Stem Cell Int (2018) 2018:5416923. 10.1155/2018/5416923 29681949 PMC5850899

[56] Pradella D Naro C Sette C Ghigna C. EMT and stemness: flexible processes tuned by alternative splicing in development and cancer progression. Mol Cancer (2017) 16(1):8. 10.1186/s12943-016-0579-2 28137272 PMC5282733

[57] McHugh D Gil J. Senescence and aging: causes, consequences, and therapeutic avenues. The J Cel Biol (2018) 217(1):65–77. 10.1083/jcb.201708092 29114066 PMC5748990

[58] Zhang DY Monteiro MJ Liu JP Gu WY. Mechanisms of cancer stem cell senescence: current understanding and future perspectives. Clin Exp Pharmacol & Physiol (2021) 48(9):1185–202. 10.1111/1440-1681.13528 34046925

[59] Xiao S Qin D Hou X Tian L Yu Y Zhang R Cellular senescence: a double-edged sword in cancer therapy. Front Oncol (2023) 13:1189015. 10.3389/fonc.2023.1189015 37771436 PMC10522834

[60] De B Morel AP Senaratne AP Ouzounova M Puisieux A. Cellular plasticity: a route to senescence exit and tumorigenesis. Cancers (2021) 13(18):4561. 10.3390/cancers13184561 34572787 PMC8468602

[61] Mosieniak G Sikora E. Polyploidy: the link between senescence and cancer. Curr Pharm Des (2010) 16(6):734–40. 10.2174/138161210790883714 20388083

[62] Sikora E Mosieniak G Sliwinska MA. Morphological and functional characteristic of senescent cancer cells. Curr Drug Targets (2016) 17(4):377–87. 10.2174/1389450116666151019094724 26477465

[63] Bharadwaj D Mandal M. Senescence in polyploid giant cancer cells: a road that leads to chemoresistance. Cytokine & Growth Factor Rev (2020) 52:68–75. 10.1016/j.cytogfr.2019.11.002 31780423

[64] Zhang S Mercado-Uribe I Xing Z Sun B Kuang J Liu J. Generation of cancer stem-like cells through the formation of polyploid giant cancer cells. Oncogene (2014) 33(1):116–28. 10.1038/onc.2013.96 23524583 PMC3844126

[65] Saleh T Carpenter VJ Bloukh S Gewirtz DA. Targeting tumor cell senescence and polyploidy as potential therapeutic strategies. Semin Cancer Biol (2022) 81:37–47. 10.1016/j.semcancer.2020.12.010 33358748 PMC8214633

[66] Zhu L Xu R Yang L Shi W Zhang Y Liu J Minimal residual disease (MRD) detection in solid tumors using circulating tumor DNA: a systematic review. Front Genet (2023) 14:1172108. 10.3389/fgene.2023.1172108 37636270 PMC10448395

[67] Alix-Panabières C Schwarzenbach H Pantel K. Circulating tumor cells and circulating tumor DNA. Annu Rev Med (2012) 63:199–215. 10.1146/annurev-med-062310-094219 22053740

[68] Wang X Wang L Lin H Zhu Y Huang D Lai M Research progress of CTC, ctDNA, and EVs in cancer liquid biopsy. Front Oncol (2024) 14:1303335. 10.3389/fonc.2024.1303335 38333685 PMC10850354

[69] Tan CR Zhou L El-Deiry WS. Circulating tumor cells versus circulating tumor DNA in colorectal cancer: pros and cons. Curr colorectal Cancer Rep (2016) 12(3):151–61. 10.1007/s11888-016-0320-y 27516729 PMC4976692

[70] Alemzadeh E Allahqoli L Dehghan H Mazidimoradi A Ghasempour A Salehiniya H. Circulating tumor cells and circulating tumor DNA in breast cancer diagnosis and monitoring. Oncol Res (2023) 31(5):667–75. 10.32604/or.2023.028406 37547763 PMC10398400

[71] Hamilton G Rath B Stickler S. Significance of circulating tumor cells in lung cancer: a narrative review. Translational Lung Cancer Res (2023) 12(4):877–94. 10.21037/tlcr-22-712 37197632 PMC10183408

[72] Calabuig-Fariñas S Jantus-Lewintre E Herreros-Pomares A Camps C. Circulating tumor cells versus circulating tumor DNA in lung cancer-which one will win? Translational Lung Cancer Res (2016) 5(5):466–82. 10.21037/tlcr.2016.10.02 27826528 PMC5099512

[73] Mondelo-Macía P García-González J León-Mateos L Castillo-García A López-López R Muinelo-Romay L Current status and future perspectives of liquid biopsy in small cell lung cancer. Biomedicines (2021) 9(1):48. 10.3390/biomedicines9010048 33430290 PMC7825645

[74] Zhang Y Zheng H Zhan Y Long M Liu S Lu J Detection and application of circulating tumor cell and circulating tumor DNA in the non-small cell lung cancer. Am J Cancer Res (2018) 8(12):2377–86. 30662798 PMC6325475

[75] Tóth K Sipos F Kalmár A Patai AV Wichmann B Stoehr R Detection of methylated SEPT9 in plasma is a reliable screening method for both left- and right-sided colon cancers. PloS one (2012) 7(9):e46000. 10.1371/journal.pone.0046000 23049919 PMC3457959

[76] Wasserkort R Kalmar A Valcz G Spisak S Krispin M Toth K Aberrant septin 9 DNA methylation in colorectal cancer is restricted to a single CpG island. BMC cancer (2013) 13:398. 10.1186/1471-2407-13-398 23988185 PMC3837632

[77] Kampel L Feldstein S Tsuriel S Hannes V Carmel Neiderman NN Horowitz G Mutated TP53 in circulating tumor DNA as a risk level biomarker in head and neck squamous cell carcinoma patients. Biomolecules (2023) 13(9):1418. 10.3390/biom13091418 37759818 PMC10527516

[78] Calapre L Giardina T Beasley AB Reid AL Stewart C Amanuel B Identification of TP53 mutations in circulating tumour DNA in high grade serous ovarian carcinoma using next generation sequencing technologies. Scientific Rep (2023) 13(1):278. 10.1038/s41598-023-27445-2 36609632 PMC9822997

[79] Hwang SH Baek SH Lee MJ Kook Y Bae SJ Ahn SG Clinical relevance of TP53 mutation and its characteristics in breast cancer with long-term follow-up date. Cancers (2024) 16(23):3899. 10.3390/cancers16233899 39682089 PMC11640694

[80] Shi C Liu S Tian X Wang X Gao P. A TP53 mutation model for the prediction of prognosis and therapeutic responses in head and neck squamous cell carcinoma. BMC cancer (2021) 21(1):1035. 10.1186/s12885-021-08765-w 34530752 PMC8447564

[81] Chiang YT Chien YC Lin YH Wu HH Lee DF Yu YL. The function of the mutant p53-R175H in cancer. Cancers (2021) 13(16):4088. 10.3390/cancers13164088 34439241 PMC8391618

[82] Lai ZY Tsai KY Chang SJ Chuang YJ. Gain-of-Function mutant TP53 R248Q overexpressed in epithelial ovarian carcinoma alters AKT-dependent regulation of intercellular trafficking in responses to EGFR/MDM2 inhibitor. Int J Mol Sci (2021) 22(16):8784. 10.3390/ijms22168784 34445495 PMC8395913

[83] Desai A Vázquez TA Arce KM Corassa M Mack PC Gray JE ctDNA for the evaluation and management of EGFR-mutant non-small cell lung cancer. Cancers (2024) 16(5):940. 10.3390/cancers16050940 38473302 PMC10930898

[84] Galizia G Lieto E Ferraraccio F De Vita F Castellano P Orditura M Prognostic significance of epidermal growth factor receptor expression in colon cancer patients undergoing curative surgery. Ann Surg Oncol (2006) 13(6):823–35. 10.1245/ASO.2006.05.052 16614884

[85] Clark GM Zborowski DM Culbertson JL Whitehead M Savoie M Seymour L Clinical utility of epidermal growth factor receptor expression for selecting patients with advanced non-small cell lung cancer for treatment with erlotinib. J Thorac Oncol official Publ Int Assoc Study Lung Cancer (2006) 1(8):837–46. 17409968

[86] Fang S Wang Z. EGFR mutations as a prognostic and predictive marker in non-small-cell lung cancer. Drug Des Dev Ther (2014) 8:1595–611. 10.2147/DDDT.S69690 25302015 PMC4189714

[87] Kanbour A Salih F Abualainin W Abdelrazek M Szabados L Al-Bozom I Leptomeningeal metastatic L858R EGFR-mutant lung cancer: prompt response to osimertinib in the absence of T790M-mutation and effective Subsequent pulsed erlotinib. OncoTargets Ther (2022) 15:659–67. 10.2147/OTT.S336012 35733652 PMC9207126

[88] Suda K Onozato R Yatabe Y Mitsudomi T. EGFR T790M mutation: a double role in lung cancer cell survival? J Thorac Oncol : official Publ Int Assoc Study Lung Cancer (2009) 4(1):1–4. 10.1097/JTO.0b013e3181913c9f 19096299

[89] Wang S Tsui ST Liu C Song Y Liu D. EGFR C797S mutation mediates resistance to third-generation inhibitors in T790M-positive non-small cell lung cancer. J Hematol & Oncol (2016) 9(1):59. 10.1186/s13045-016-0290-1 27448564 PMC4957905

[90] Beganovic S. Clinical significance of the KRAS mutation. Bosnian J Basic Med Sci (2009) 9(Suppl. 1):S17–S20. 10.17305/bjbms.2009.2749 19912113 PMC5655166

[91] Perets R Greenberg O Shentzer T Semenisty V Epelbaum R Bick T Mutant KRAS circulating tumor DNA is an accurate tool for pancreatic cancer monitoring. The oncologist (2018) 23(5):566–72. 10.1634/theoncologist.2017-0467 29371474 PMC5947453

[92] Ernst SM van Marion R Atmodimedjo PN de Jonge E Mathijssen RHJ Paats MS Clinical utility of circulating tumor DNA in patients with advanced KRASG12C-mutated NSCLC treated with sotorasib. J Thorac Oncol official Publ Int Assoc Study Lung Cancer (2024) 19(7):995–1006. 10.1016/j.jtho.2024.04.007 38615940

[93] Tang D Kang R. Glimmers of hope for targeting oncogenic KRAS-G12D. Cancer Gene Ther (2023) 30(3):391–3. 10.1038/s41417-022-00561-3 36414681

[94] Stanland LJ Huggins HP Sahoo SS Porrello A Chareddy Y Azam SH A first-in-class EGFR-directed KRAS G12V selective inhibitor. Cancer cell (2025), S1535-6108. 10.1016/j.ccell.2025.05.016 40541192 PMC12230742

[95] Qunaj L May MS Neugut AI Herzberg BO. Prognostic and therapeutic impact of the KRAS G12C mutation in colorectal cancer. Front Oncol (2023) 13:1252516. 10.3389/fonc.2023.1252516 37790760 PMC10543081

[96] Sobczuk P Kozak K Kopeć S Rogala P Świtaj T Koseła-Paterczyk H The use of ctDNA for BRAF mutation testing in routine clinical practice in patients with advanced melanoma. Cancers. (2022) 14(3):777. 10.3390/cancers14030777 35159044 PMC8833667

[97] Klein-Scory S Baraniskin A Schmiegel W Mika T Schroers R Held S Evaluation of circulating tumor DNA as a prognostic and predictive biomarker in BRAF V600E mutated colorectal cancer-results from the FIRE-4.5 study. Mol Oncol (2025) 19(2):344–56. 10.1002/1878-0261.13778 39630848 PMC11793001

[98] Bharti J Gogu P Pandey SK Verma A Yadav JP Singh AK BRAF V600E in cancer: exploring structural complexities, mutation profiles, and pathway dysregulation. Exp Cel Res (2025) 446(1):114440. 10.1016/j.yexcr.2025.114440 39961465

[99] Zengarini C Mussi M Veronesi G Alessandrini A Lambertini M Dika E. BRAF V600K vs. BRAF V600E: a comparison of clinical and dermoscopic characteristics and response to immunotherapies and targeted therapies. Clin Exp Dermatol (2022) 47(6):1131–6. 10.1111/ced.15113 35080260 PMC9311196

[100] Dumbrava EE Call SG Huang HJ Stuckett AL Madwani K Adat A PIK3CA mutations in plasma circulating tumor DNA predict survival and treatment outcomes in patients with advanced cancers. ESMO open (2021) 6(5):100230. 10.1016/j.esmoop.2021.100230 34479035 PMC8414046

[101] Cizkova M Susini A Vacher S Cizeron-Clairac G Andrieu C Driouch K PIK3CA mutation impact on survival in breast cancer patients and in ERα, PR and ERBB2-based subgroups. Breast Cancer Res BCR (2012) 14(1):R28. 10.1186/bcr3113 22330809 PMC3496146

[102] Dumont AG Dumont SN Trent JC. The favorable impact of PIK3CA mutations on survival: an analysis of 2587 patients with breast cancer. Chin J Cancer (2012) 31(7):327–34. 10.5732/cjc.012.10032 22640628 PMC3777497

[103] Samuels Y Waldman T. Oncogenic mutations of PIK3CA in human cancers. Curr Top Microbiol Immunol (2010) 347:21–41. 10.1007/82_2010_68 20535651 PMC3164550

[104] Guo S Loibl S von Minckwitz G Darb-Esfahani S Lederer B Denkert C. PIK3CA H1047R mutation associated with a lower pathological complete response rate in triple-negative breast cancer patients treated with anthracycline-taxane-based neoadjuvant chemotherapy. Cancer Res Treat (2020) 52(3):689–96. 10.4143/crt.2019.497 32019278 PMC7373870

[105] Leontiadou H Galdadas I Athanasiou C Cournia Z. Insights into the mechanism of the PIK3CA E545K activating mutation using MD simulations. Scientific Rep (2018) 8(1):15544. 10.1038/s41598-018-27044-6 30341384 PMC6195558

[106] Keraite I Alvarez-Garcia V Garcia-Murillas I Beaney M Turner NC Bartos C PIK3CA mutation enrichment and quantitation from blood and tissue. Scientific Rep (2020) 10(1):17082. 10.1038/s41598-020-74086-w 33051521 PMC7555501

[107] Shen N Wang T Li D Zhu Y Xie H Lu Y. Hypermethylation of the SEPT9 gene suggests significantly poor prognosis in cancer patients: a systematic review and meta-analysis. Front Genet (2019) 10:887. 10.3389/fgene.2019.00887 31608117 PMC6761278

[108] Lu P Zhu X Song Y Luo Y Lin J Zhang J Methylated septin 9 as a promising biomarker in the diagnosis and recurrence monitoring of colorectal cancer. Dis markers (2022) 2022:7087885. 10.1155/2022/7087885 35818587 PMC9271001

[109] Song L Li Y. SEPT9: a specific circulating biomarker for colorectal cancer. Adv Clin Chem (2015) 72:171–204. 10.1016/bs.acc.2015.07.004 26471083

[110] Ratajska M Koczkowska M Żuk M Gorczyński A Kuźniacka A Stukan M Detection of BRCA1/2 mutations in circulating tumor DNA from patients with ovarian cancer. Oncotarget (2017) 8(60):101325–32. 10.18632/oncotarget.20722 29254167 PMC5731877

[111] Zhu Y Wu J Zhang C Sun S Zhang J Liu W BRCA mutations and survival in breast cancer: an updated systematic review and meta-analysis. Oncotarget (2016) 7(43):70113–27. 10.18632/oncotarget.12158 27659521 PMC5342539

[112] Borg A Haile RW Malone KE Capanu M Diep A Törngren T Characterization of BRCA1 and BRCA2 deleterious mutations and variants of unknown clinical significance in unilateral and bilateral breast cancer: the WECARE study. Hum Mutat (2010) 31(3):E1200–40. 10.1002/humu.21202 20104584 PMC2928257

[113] Yi Z Feng K Lv D Guan Y Shao Y Ma F Genomic landscape of circulating tumor DNA in HER2-low metastatic breast cancer. Signal Transduction Targeted Ther (2024) 9:345. 10.1038/s41392-024-02047-0 39648226 PMC11625825

[114] Reix N Malina C Chenard MP Bellocq JP Delpous S Molière S A prospective study to assess the clinical utility of serum HER2 extracellular domain in breast cancer with HER2 overexpression. Breast Cancer Res Treat (2016) 160(2):249–59. 10.1007/s10549-016-4000-z 27709352 PMC5065601

[115] Shin JW Kim S Ha S Choi B Kim S Im SA The HER2 S310F mutant can form an active heterodimer with the EGFR, which can Be inhibited by cetuximab but not by trastuzumab as well as pertuzumab. Biomolecules (2019) 9(10):629. 10.3390/biom9100629 31635022 PMC6843359

[116] Li J Xiao Q Bao Y Wang W Goh J Wang P HER2-L755S mutation induces hyperactive MAPK and PI3K-mTOR signaling, leading to resistance to HER2 tyrosine kinase inhibitor treatment. Cell Cycle (Georgetown, Tex.). (2019) 18(13):1513–22. 10.1080/15384101.2019.1624113 31135266 PMC6592242

[117] Kahana-Edwin S McCowage G Cain L Saletta F Yuksel A Graf N Exploration of CTNNB1 ctDNA as a putative biomarker for hepatoblastoma. Pediatr Blood & Cancer (2020) 67(11):e28594. 10.1002/pbc.28594 32881242

[118] Lu G Lin J Song G Chen M. Prognostic significance of CTNNB1 mutation in hepatocellular carcinoma: a systematic review and meta-analysis. Aging (2023) 15(18):9759–78. 10.18632/aging.205047 37733676 PMC10564414

[119] Rebouissou S. Genotype-phenotype correlation of CTNNB1 mutations reveals different ß-catenin activity associated with liver tumor progression. Hepatol Baltimore, Md (2016) 64(6):2047–61. 10.1002/hep.28638 27177928

[120] Ding X Yang Y Han B Du C Xu N Huang H Transcriptomic characterization of hepatocellular carcinoma with CTNNB1 mutation. PloS one (2014) 9(5):e95307. 10.1371/journal.pone.0095307 24798046 PMC4010419

[121] Kong SL Liu X Tan SJ Tai JA Phua LY Poh HM Complementary sequential circulating tumor cell (CTC) and cell-free tumor DNA (ctDNA) profiling reveals metastatic heterogeneity and genomic changes in lung cancer and breast cancer. Front Oncol (2021) 11:698551. 10.3389/fonc.2021.698551 34336686 PMC8322849

[122] Koyanagi K Mori T O'Day SJ Martinez SR Wang HJ Hoon DSB. Association of circulating tumor cells with serum tumor-related methylated DNA in peripheral blood of melanoma patients. Cancer Res (2006) 66(12):6111–7. 10.1158/0008-5472.CAN-05-4198 16778184 PMC2856454

[123] Shaw JA Brown J Coombes RC Jacob J Payne R Lee B Circulating tumor cells and plasma DNA analysis in patients with indeterminate early or metastatic breast cancer. Biomarkers Med (2011) 5(1):87–91. 10.2217/bmm.10.118 21319970

[124] Matuschek C Bölke E Lammering G Gerber PA Peiper M Budach W Methylated APC and GSTP1 genes in serum DNA correlate with the presence of circulating blood tumor cells and are associated with a more aggressive and advanced breast cancer disease. Eur J Med Res (2010) 15(7):277–86. 10.1186/2047-783x-15-7-277 20696638 PMC3351951

[125] Wu CY Lee CL Fu JY Yang CT Wen CT Circulating tumor cells as a tool of minimal residual disease can predict lung cancer recurrence: a longitudinal, prospective trial. Diagnostics (Basel, Switzerland) (2020) 10(3):144. 10.3390/diagnostics10030144 32155787 PMC7151004

[126] Li W Zhang X Yang Y Lin J Zhou K Sun R Circulating tumor cells are a good predictor of tumor recurrence in clinical patients with gastric cancer. Scientific Rep (2024) 14(1):12758. 10.1038/s41598-024-63305-3 38830909 PMC11148116

[127] Kaifi JT Li G Clawson G Kimchi ET Staveley-O'Carroll KF. Perioperative circulating tumor cell detection: current perspectives. Cancer Biol & Ther (2016) 17(8):859–69. 10.1080/15384047.2016.1167296 27045201 PMC5004694

[128] Efthymiou V Queenan N Haas M Naegele S Goss D Faden DL. Circulating tumor DNA in the immediate post-operative setting. medRxiv: preprint server Health Sci (2023):2023.09.30.23296390. 10.1101/2023.09.30.23296390 37873394 PMC10593016

[129] Wang Z Bai J Jiang D Li Y Hu X Efetov S Liquid biopsy for monitoring minimal residual disease in colorectal cancer: a promising approach with clinical implications. Clin Surg Oncol (2024) 3(3):100056. 10.1016/j.cson.2024.100056

[130] Radovich M Jiang G Hancock BA Chitambar C Nanda R Falkson C Association of circulating tumor DNA and circulating tumor cells after neoadjuvant chemotherapy with disease recurrence in patients with triple-negative breast cancer: preplanned secondary analysis of the BRE12-158 randomized clinical trial. JAMA Oncol (2020) 6(9):1410–5. 10.1001/jamaoncol.2020.2295 32644110 PMC7349081

[131] Sharma P Goswami S Raychaudhuri D Siddiqui BA Singh P Nagarajan A Immune checkpoint therapy-current perspectives and future directions. Cell (2023) 186(8):1652–69. 10.1016/j.cell.2023.03.006 37059068

[132] Horn LA Fousek K Palena C. Tumor plasticity and resistance to immunotherapy. Trends Cancer (2020) 6(5):432–41. 10.1016/j.trecan.2020.02.001 32348738 PMC7192950

[133] Benboubker V Boivin F Dalle S Caramel J. Cancer cell phenotype plasticity as a Driver of immune escape in melanoma. Front Immunol (2022) 13:873116. 10.3389/fimmu.2022.873116 35432344 PMC9012258

[134] Qin S Jiang J Lu Y Nice EC Huang C Zhang J Emerging role of tumor cell plasticity in modifying therapeutic response. Signal Transduction Targeted Therapy (2020) 5(1):228. 10.1038/s41392-020-00313-5 33028808 PMC7541492

[135] da Silva-Diz V Lorenzo-Sanz L Bernat-Peguera A Lopez-Cerda M Muñoz P. Cancer cell plasticity: impact on tumor progression and therapy response. Semin Cancer Biol (2018) 53:48–58. 10.1016/j.semcancer.2018.08.009 30130663

[136] Niu X Liu W Zhang Y Liu J Zhang J Li B Cancer plasticity in therapy resistance: mechanisms and novel strategies. Drug Resist Updates (2024) 76:101114–7646. 10.1016/j.drup.2024.101114 38924995

[137] Lisencu LA Bonci EA Irimie A Balacescu O Lisencu C. The role of circulating tumor cells in chemoresistant metastatic breast cancer. J Clin Med (2021) 10(4):684. 10.3390/jcm10040684 33578862 PMC7916545

[138] Yadav P Rajendrasozhan S Lajimi RH Patel RR Heymann D Prasad NR. Circulating tumor cell markers for early detection and drug resistance assessment through liquid biopsy. Front Oncol (2025) 15:1494723. 10.3389/fonc.2025.1494723 40260304 PMC12009936

[139] Mladinich M Ruan D Chan CH. Tackling cancer stem cells via inhibition of EMT transcription factors. Stem Cell Int (2016) 2016:5285892. 10.1155/2016/5285892 27840647 PMC5093281

[140] Smit MA Peeper DS. Epithelial-mesenchymal transition and senescence: two cancer-related processes are crossing paths. Aging (2010) 2(10):735–41. 10.18632/aging.100209 20975209 PMC2993803

[141] Faheem MM Seligson ND Ahmad SM Rasool RU Gandhi SG Bhagat M Convergence of therapy-induced senescence (TIS) and EMT in multistep carcinogenesis: current opinions and emerging perspectives. Cell Death Discov (2020) 6:51. 10.1038/s41420-020-0286-z 32566256 PMC7295779

[142] Mu P Zhang Z Benelli M Karthaus WR Hoover E Chen CC SOX2 promotes lineage plasticity and antiandrogen resistance in TP53- and RB1-deficient prostate cancer. Science (New York, N.Y.) (2017) 355(6320):84–8. 10.1126/science.aah4307 28059768 PMC5247742

[143] Richard G Dalle S Monet MA Ligier M Boespflug A Pommier RM ZEB1-mediated melanoma cell plasticity enhances resistance to MAPK inhibitors. EMBO Mol Med (2016) 8(10):1143–61. 10.15252/emmm.201505971 27596438 PMC5048365

[144] Beltran H Prandi D Mosquera JM Benelli M Puca L Cyrta J Divergent clonal evolution of castration-resistant neuroendocrine prostate cancer. Nat Med (2016) 22:298–305. 10.1038/nm.4045 26855148 PMC4777652

[145] Bao-Caamano A Costa-Fraga N Cayrefourcq L Rodriguez-Casanova A Muinelo-Romay L López-López R Epigenomic reprogramming of therapy-resistant circulating tumor cells in colon cancer. Front Cel Dev Biol (2023) 11:1291179. 10.3389/fcell.2023.1291179 38188020 PMC10771310

[146] Gatenby RA Silva AS Gillies RJ Frieden BR. Adaptive therapy. Cancer Res (2009) 69(11):4894–903. 10.1158/0008-5472.CAN-08-3658 19487300 PMC3728826

[147] Zhang L Ma J Liu L Li G Li H Hao Y Adaptive therapy: a tumor therapy strategy based on Darwinian evolution theory. Crit Rev oncology/hematology (2023) 192:104192. 10.1016/j.critrevonc.2023.104192 37898477

